# Reviewing the Itaewon Halloween crowd crush, Korea 2022: Qualitative content analysis

**DOI:** 10.12688/f1000research.135265.1

**Published:** 2023-07-13

**Authors:** Kyoo-Man Ha

**Affiliations:** 1 Faculty of Resilience, Rabdan Academy, Abu Dhabi, 114646, United Arab Emirates

**Keywords:** emergency planning; ordinary events; contingency planning; special events; inclusion; governments; tourists; emergency training

## Abstract

Background: The issue of crowd crushes has been not only very complicated but also uncertain. This article aimed to evaluate how situations such as the Itaewon Halloween crowd crush in South Korea in 2022 can be better managed to reduce human loss. Methods: Qualitative analysis was the key methodology used to compare emergency planning for ordinary events with contingency planning for special events, focusing on four stakeholders, namely governments, businesses, voluntary organizations, and other local communities. Results: The key finding was that all stakeholders would need to supplement emergency planning for ordinary events with contingency planning for special events for the nation. They must embody cooperation, cutting-edge technologies, routinized updates, situation awareness, political rationality, training and exercise, and others, based on inclusion. Conclusions: This is a pioneer study that examined the Itaewon crowd crush more comprehensively than others in particular by including many disaster management principles.

## Introduction

The Itaewon crowd crush occurred in South Korea (hereinafter KR) during the Halloween festivities on October 29, 2022. Itaewon is located in the Yongsan-gu district of Seoul. It is known as a foreigner-friendly area owing to its trendy restaurants, nightclubs, and nightlife. On October 29, 2022, many young adults gathered in a downhill alley measuring 40 meters long and 4 meters wide, to celebrate Halloween. As the alley filled with people, they could not move and thus had no choice but to stand shoulder-to-shoulder, slowly suffocating. This resulted in the death of 158 people (i.e., 132 Koreans and 26 foreigners), with 196 others injured ( Lee *et al.*, 2022).

The vast majority of Koreans expressed their sadness at the national tragedy, but a few stakeholders attempted to take inappropriate actions. For example, some policymakers refused to use the term “Itaewon crowd crush” and instead called it the 10.29 disaster ( Kim, 2022). In a sense, they refused to claim responsibility by hiding behind a new name. Also, by giving it a name that does not include Itaewon, they have tried to decrease the extent of stigma attached to Itaewon as a tragic area. Another similar example is the case of the 9/11 terror attacks. Clapp (2010) suggests the name of the 9/11 terror attacks was not intended to avoid the stigma attached to New York City; rather, 911 is the emergency phone number in the United States.

Multiple experts have scientifically analyzed the Itaewon crowd crush from different perspectives. According to one of them, although KR requires restrictive safety protocols for official events, the same requirement does not apply to public spaces where a large number of people gather informally ( Lee, 2022b). Because of such ambiguous safety measures, not a single public agency promptly claimed related responsibilities to take charge of such a massive crowd. Similarly, high-ranking officials admitted that the nation did not have any emergency plan to manage such events without official organizers ( Loh, 2022). Thus, a clear gap exists between the emergency plans and the emerging reality in KR.

When focusing on emergency planning for ordinary events, the nation did not equally consider contingency planning for special events ( Choi *et al.*, 2022: 1-10). In fact, many professionals in the field of emergency management are not aware of the differences between the two types of planning. Further, the Korean research field has not delved into crowd crush as a major research topic. Many Koreans have come to wonder what their governments have in place for managing crowd crushes. Thus, the research question guiding this study is as follows: What is the most effective way to prevent situations such as the Itaewon Halloween crowd crush in KR?

The objective of this research has been to review how to deal with situations similar to the Itaewon crowd crush in order to decrease human loss. This study compared emergency planning for ordinary events with contingency planning for special events with a focus on four analytical units, namely governments, businesses, voluntary organizations, and other local communities. The key finding is that all stakeholders must try to supplement emergency planning for ordinary events with contingency planning for special events. Further, they must address not only inclusion but also cooperation, time-efficiency, advanced technologies, public awareness, political rationality, education and training, and others.

## Literature review

Crowd crush is a catastrophe involving a body of moving people packed tightly within a limited space (or dangerously overcrowded), resulting in mass deaths from compressive asphyxia. Three kinds of group motivation have been suggested as the major cause of crowd crush: traffic interruption, flight, and craze ( Raineri, 2004: 1-14). A few cases of crowd crush resulting from traffic interruption have been reported in the past. Other cases of crowd crush result from the high extent of an individual’s tendency to choose flight, which includes the desire to run away, escape, or avoid. The remaining incidents of crowd crush happen due to mass craze while rushing to obtain a common objective.

For effective crowd crush management, all stakeholders in the field of emergency management must respond to the needs of mass crowds with respect and sensitivity ( Carter *et al.*, 2018: 937-940). Without understanding the specific needs of mass crowds, it would be almost difficult to improve catastrophic situations in time. Emergency responders must openly and keenly communicate with mass crowds for their compliance, in particular before and during crowd crush. To this end, major stakeholders must sustain various guidelines and strategies for the management of potential crowd crushes.

Johari’s window for interpersonal awareness includes four areas: the open area, the hidden area, the blind area, and the unknown area ( Luft, 1961: 6-7). Within this context, the Itaewon crowd crush should be included in the blind area. As the Korean government showed its inefficiency in managing unexpected events such as the Itaewon crowd crush, many practitioners in foreign countries seriously discussed strategies related to crowd management. In other words, situations such as the Itaewon crowd crush were not anticipated in KR, but other countries are aware of it and have strategies in place. Therefore, the tragedy falls in the blind area of the Johari window.

Several similar tragedies have transpired in the past across the globe ( Johnston, 2022). For instance, 11 young students lost their lives while trying to enter a The Who concert in Cincinnati, Ohio, in December 1979. Similarly, 97 soccer fans were killed in Sheffield, the United Kingdom, in April 1989, due to a bottleneck phenomenon. About 2,400 pilgrims were killed near the city of Mecca, Saudi Arabia, during the Hajj pilgrimage in September 2015. Approximately 130 soccer fans were killed in Malang, Indonesia, during a mass stampede in October 2022.

Potential crowd crush situations, in particular during the post-covid period, were recently predicted by a few professionals ( Morton and Power, 2022: 1-9). The pandemic has impacted the daily lives of almost all individuals around the world. Against the backdrop of social distancing, lockdown, travel constraints, and others, so many people have been willing to gather together in public places. Despite COVID-19 variants or sub-variants still circulating in the air, people are now trying to relieve their mental stress or psychological impact by participating in crowd gatherings. This has increased the likelihood of crowd crush.

There are two kinds of events according to the field of emergency management: ordinary events and special events. Ordinary events have four distinctive characteristics ( Mair and Whitford, 2013: 6-8; Richards *et al.*, 2022: 1-22). First, ordinary events are organized and held in various local communities on a regular basis; in other words, they are routine events. Second, ordinary events do not require many resources. Third, although ordinary events involve a number of people, they are recurring events. Fourth, ordinary events do not require special permits. Examples include private parties, professional baseball games, political conventions, workshops, and others.

On the other hand, special events have four distinct elements that characterize them ( FEMA, 2013). They are non-routine events held in a local community. Special events put a substantial strain or pressure on local resources. Further, they generally involve a significant number of people. In addition, special events require local agencies to work on additional emergency mitigation, emergency preparedness, and emergency planning. Examples include spontaneous events, controversial political events, regional competitions, and others.

However, it is not easy for even experts to determine if a specific event such as the Itaewon Halloween party must be categorized as an ordinary event or a special event. As such, political rallies routinely occur in various democratic countries, but nobody knows exactly when they will be held. A local community does not have the same number of resources as other communities. The number of people attending such events depends on several criteria. Also, additional emergency mitigation, preparedness, and planning usually entail complicated processes.

Plans are a set of targeted actions carried out to achieve a goal; in other words, planning is a lasting process that leads to goal fulfillment ( HACCP Mentor, 2018). Also, while planning is the whole procedure of developing a series of plans, many kinds of planning are available in the field of emergency management. Two kinds of planning are chosen and discussed in this research paper, namely emergency planning versus contingency planning, to analyze the relationship between ordinary events and special events.

To elaborate, an emergency is a specific situation, the emergency plan entails what individuals or institutions are expected to do in the event of diverse emergencies. On the other hand, when an event does not go forward as planned, related incidents or accidents occur that are beyond the control of stakeholders. At this time, a contingency plan is implemented depending on the circumstances. In summary, emergency planning is related to setting up a usual plan(s) for ordinary events, whereas contingency planning includes the provision of a revised plan(s) for dealing with the worst-case scenario, or a Plan B for special events.

The subject of crowd crush and related management has been researched extensively in the international community ( Almutairi *et al.*, 2022: 1-13). Various research areas have examined the issue, such as disaster management, safety science, medical science, transportation, tourism, physics, computer science, economics, laws, police science, geography, and others, each sharing their perspective on the matter and providing tailored management alternatives. For instance, Tracy H. Pearl studied the legal aspects of crowd crush in the United States ( Pearl, 2015: 1-45). According to Pearl, although crowd crushes have occurred across a vast range of places and situations in the US, the nation has often failed to protect its citizens from the aftermath. In other words, statutory laws in the United States left the majority of Americans unprotected from crowd crush. In particular, crowd crush was hardly litigated, as being almost based on common law (or negligence claims). Hence, this paper suggests that the courts refer to jurisprudence of crowd crush by relying on modern science.

During the last decade, a number of researchers systematically examined crowd simulation, in particular as computational resources (including artificial intelligence) dramatically increased. A group of researchers delved into crowd behaviors, movements, and psychology, while the other group focused on various disasters including fires, floods, and earthquakes. George Sidiropoulos *et al.* also researched the subject of crowd simulation ( Sidiropoulos *et al.*, 2020: 1-23).

P.S. Karthika *et al.* examined the issue of accessibility to religious mass gatherings ( Karthika *et al.*, 2022: 1-18). They assumed that the level of crowd management in a certain situation was strongly influenced by the behavior of various pedestrians. They confirmed that pedestrians would like to choose their favorite routes on the basis of not only distance to a particular destination but also the direction. Among many, these researchers recommended the importance of short-term road closures to prevent the occurrence of population influx into mass pilgrimages.

Research on crowd crush or related accident management in KR has not been quite robust ( Kim *et al.*, 2021: 8-10). Only a few researchers have examined the subject, such as an investigation into the crowd crush at the Sangju music concert in 2005, which was a study on evacuation velocity. In addition, while making all their efforts to deal with frequent disasters in the nation, such as typhoons, floods, and building fires, not only researchers but also government officials have failed to consider how to wisely cope with crowd crush.

Taking the above all into consideration, this paper presents a systematic review of KR’s Itaewon crowd crush as a pioneering study. This paper analyzed the subject more comprehensively than previous Korean studies on the matter. Similarly, this study applied many disaster management principles suggested by international researchers, to the case in point.

## Methods

The methodology used in this research work here is qualitative content analysis in systematic reviews. In general, systematic reviews address answerable and specific questions, while trying to diagnose the key issue and then develop an alternative plan ( Campbell *et al.*, 2015: 154-160). The analysis was divided into four steps: planning the research structure, collecting qualitative text data, analyzing qualitative text data, and presenting qualitative text data ( Qualtrics, 2022).

**Figure 1.**
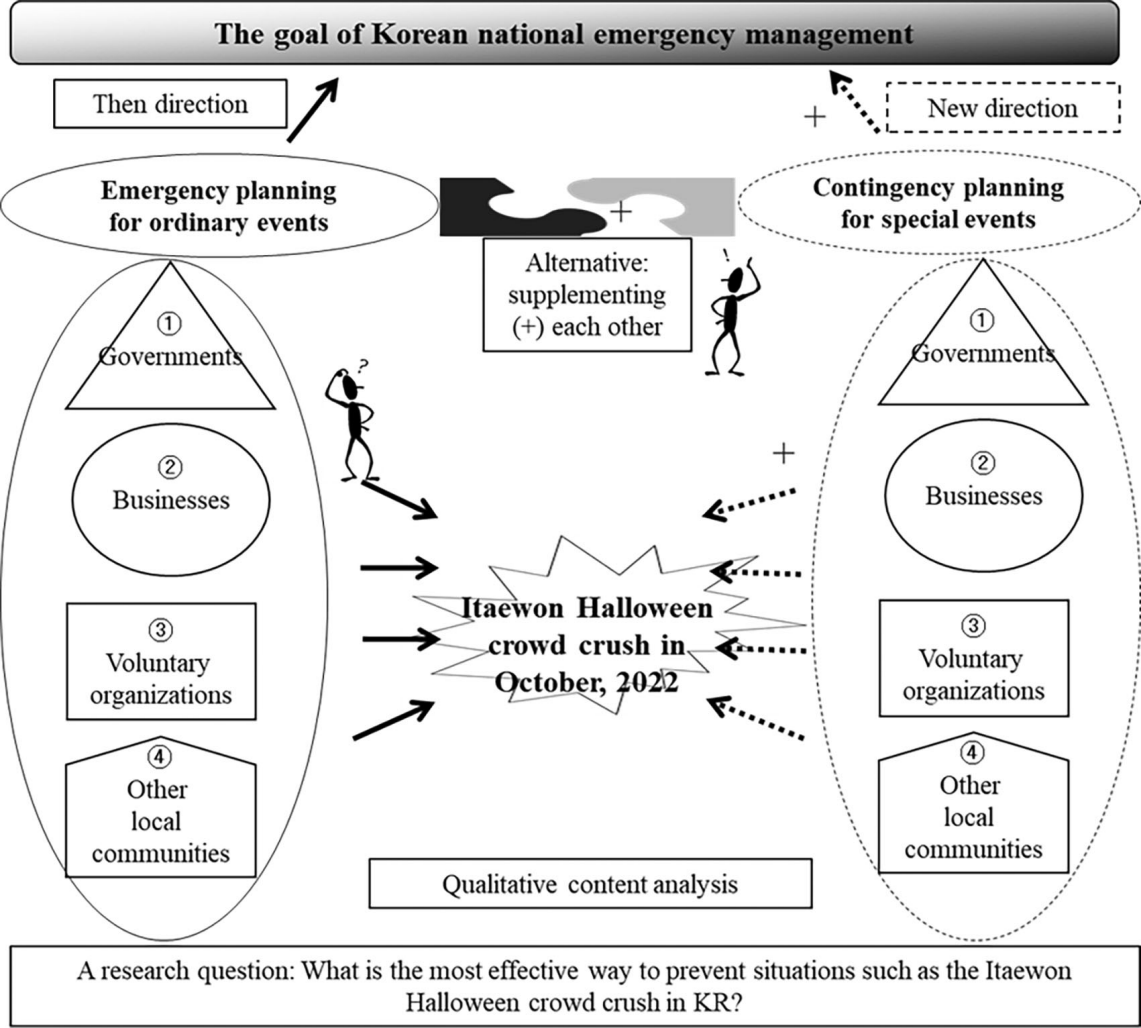
Research structure.

Figure 1 shows the research structure to include a research question, units of analysis, thematic categories, and others. The research has allocated four analytical units such as governments, businesses, voluntary organizations, and other local communities. These four units have been traditionally treated as key stakeholders or players in the field of emergency management ( FEMA, 2021). Two thematic categories included are emergency planning for ordinary events versus contingency planning for special events. In fact, those two thematic categories comprehensively combine afore-mentioned four analytical units.

### Information sources and search strategy

For collecting qualitative text data, several search engines were utilized, such as ScienceDirect, Oxford University Press, ProQuest, EBSCO, and Google.com, among others. The keywords used include “contingency planning for special events,” “ordinary events vs. special events,” “crowd management,” “crowd crush vs. stampede,” “emergency planning vs. contingency planning,” “crowd management in Korea,” and others. ScienceDirect has many research articles on crowd management, while Google has not only multiple research articles on emergency planning vs. contingency planning but also government documents for guidance on contingency planning.

The identification criterion (or criterion of qualitative data inclusion or exclusion) was if the article is related to crowd management, emergency planning, and contingency planning. Those three phrases have well reflected various aspects of this research. At the same time, identified articles or documents have clearly stated the goal of this paper with pre-designed eligibility. To elaborate, when the article includes appropriate contents on crowd management, emergency planning, and contingency planning, it has been included into this research. On the other hand, if the article does not discuss many contents on those three factors, it has been surely excluded here.

### Study selection, data extraction and analysis

The second half of the methodological process has certain characteristic features. During the analysis of qualitative text data, coding (which is to label qualitative data to identify analytical themes) has been carried out by substantially organizing the same four analytical units into two thematic categories ( Delve and Limpaecher, 2023). In doing so, the research has made efforts to flexibly interpret qualitative text data while checking if related text supports the research structure. This research has tried to identify and draw appropriate patterns in the results.

As a similar token, related source selection process relied on two reviewers such as an emergency manager (i.e., the author) and a graduate student (as a research volunteer). They reviewed qualitative text data independently ( PRISMA, 2023). Hence, their feedback has been respectively reflected to the source selection process. In addition, synthesis is to combine and then evaluate identified or extracted texts, while paying attention to textual descriptions. In terms of these synthesis methods, the comparative perspective was flexibly used such as comparing against each reviewer’s synthesis. The comparative perspective was based on the fact that synthesis methods could not be fully implemented without comparing appropriate viewpoints ( Fachelli and Lopez-Roldan, 2021: 757–761). In other words, this research tried to systematically compare an emergency manager’s selected sources and a graduate student’s selected sources, not to miss any important text. Two reviewers often tried to see if a specific text would have an appropriate level of evidence on the subject. They continued to assess validity and quality of identified evidence. Not one but two reviewers could increase the number of relevant documents for this research in particular through screening process. When common resources were identified, the research willingly adopted them. Two reviewers discussed differences between them and then decided to include or exclude a specific resource.

## Results

Regarding the results of search and selection process, several types of qualitative data have been identified in this paper such as research articles (21), books (3), government documents (4), and websites (13). The number of all qualitative data has been 41. At the same time, those qualitative data have been identified via several search engines to include Google.com (11), Oxford University Press (3), ScienceDirect (7), EBSCO (3), ProQuest (2), and other websites (15). Some qualitative data have been identified over two or more search engines. At any rate, reporting results based on PRISMA is to increase not only transparency but also accuracy of systematic reviews ( Page *et al.*, 2022: 156-159).

### Emergency planning for ordinary events

*Governments*

The Basic Plan on National Safety Management (BPNSM) has let the Ministry of the Interior and Safety (MOIS) take charge of safety and emergency management at the national level. At the local level, local policemen around Itaewon are supposed to deal with mass crowds with the support of local firefighters and local government employees ( MOIS, 2023). Nonetheless, only a few officials were aware of the potential danger of public gatherings around Itaewon; thus, the local community failed to decisively deal with it. According to the minister of MOIS, the police did not have enough emergency responders to deal with the Itaewon crowd crush because they had to manage other political rallies.

*Businesses*

Many local businesses were located around the anonymous alley within Itaewon, while fully or partially providing emergency services and products for residents or visitors. Nonetheless, the alley was narrower than expected in terms of space because of unlawful construction around those businesses. For instance, the Hamilton Hotel had illegally extended its building toward the alley. Some other small businesses, such as cuisine restaurants, dancing clubs, drinking bars, and shops renovated or extended their buildings, without following the Korean building code. These illegal activities partially contributed to the bottleneck phenomenon in Itaewon.

*Voluntary organizations*

Just during or after the occurrence of the Itaewon crowd crush, quite a few spontaneous volunteers played an important role in responding to it ( Park, 2022). Without their voluntary activities such as cardiopulmonary resuscitation (CPR), the casualty number would have been bigger. They also attempted to translate what foreign visitors asked for while cleaning up debris from the emergency spot. Similarly, some business owners volunteered to provide their business spaces or services for carrying out disaster relief. Notwithstanding, voluntary activities were limited to the phases of emergency response/recovery.

*Other local communities*

Other local communities, including mass media, local residents, tourists, and others, did not pay much attention to the mass crowd gathering around Itaewon during Halloween parties. Although some mass media reported the popularity of Halloween parties in the nation, they did not seriously discuss the potential risks of mass crowds at all. Thus, not only local residents but even tourists were uninformed of the potential danger around Itaewon. In summary, the local communities did not have an understanding of personal safety during Itaewon Halloween parties.

### Contingency planning for special events

*Governments*

All government employees should have approached the Itaewon crowd how they approach political rallies in the region. Even though the Itaewon even did not have any formal organizer, such incidents must be included in the government’s emergency management protocol. Therefore, the central government needs to set up the Korean version of the National Response Framework (NRF) beyond the BPNSM. Appropriate roles and responsibilities must be assigned to each stakeholder before the occurrence of special events. For example, local policemen being the front line of defense must control unexpected crowds in time. As supporting institutions, not only local government officials but also local firefighters must directly or indirectly help local policemen around mass crowds.

*Businesses*

Various local businesses around Itaewon need to address the issue of structural safety (or load capacities) as much as is feasible to prevent recurrence ( Alkhadim *et al.*, 2018: 26-28). As spectator safety is important during special events, local businesses must consider the safety of their structures, including not only building structures but also temporary platform structures. Similarly, businesses must continue to implement spot improvements to reduce localized bottleneck phenomena. Further, all structures around local businesses must entirely comply with the Korean building code.

*Voluntary organizations*

Voluntary organizations can recruit volunteers to expand their activities into the phase of emergency preparedness, as well as the phases of emergency response/recovery regarding special events. This can prevent another crowd crush tragedy. Thus, emergency training and exercises need to provide information to the affiliated volunteers on how to prepare for special events (e.g., crowd monitoring, emergency warning, etc.). Emergency education should spread the knowledge on preparedness to schools, community organizations, and potential spontaneous volunteers.

*Other local communities*

Mass media must spread information on the danger of special events to residents, tourists, and others during the phase of emergency preparedness, without mentioning reports of emergency response/recovery ( Eriksson, 2018: 11-12). Mass media may create, disseminate, and even educate all individuals on the danger of mass crowds in advance while considering crowd personalities. Vice versa, by recognizing that personal safety is one of the most important strategies in the field, all residents and tourists must open their communication channels with media advisories, emergency alerts, and other safety issues.

## Implications

Emergency planning for ordinary events does not have a unilateral relationship but a reciprocal relationship with contingency planning for special events ( Koenti, 2022: 1-9). Strictly speaking, the fundamental principles of emergency planning for ordinary events are very similar (or even the same) to that of contingency planning for special events, but the latter is slightly bigger than the former in terms of its scope. Thus, emergency planning for ordinary events should not be replaced by contingency planning for special events but supplemented with the latter.

The goal of emergency management may not be smoothly achieved unless the needs of all people are considered when managing its scope ( Sheehy *et al.*, 2022: 1-17). When marginalized people suffer from the impact of multiple disasters far more than prosperous people, the needs of marginalized people must be equally addressed in the name of diversity. Without substantial inclusion, the field of emergency management cannot reduce the physical or social impacts of disasters.

The fact that KR needs to supplement its emergency planning for ordinary events with contingency planning for special events strongly indicates that the nation must embody inclusion to the fullest extent. Management of events involving unexpected crowds or mass crowds without organizers has not been equally included in the Korean field of emergency management yet. To date, the group of mass crowd used to belong to the group of marginalized people in KR. As Korean society develops, the nation must address the concept of explicit and fair inclusion in the emergency management field.

Further, various stakeholders must cooperate to achieve successful supplementation ( Persson and Granberg, 2021: 1336-1338). When a few stakeholders fail to manage crowd crush, cooperation with other stakeholders is strongly recommended. However, cooperation based on communication among multiple stakeholders incurs significant costs and realizing that coordination with multiple motivations and resources is stressful and challenging. Notwithstanding, cooperation can overcome differences among individuals, organizations, local cultures, and other politics.

The findings of this research contribute to expanding the scope of emergency management literature in KR. The Korean research field includes just emergency planning for ordinary events in its scope. However, this research provides findings to include not only emergency planning for ordinary events but also contingency planning for special events in the research scope. On a greater scale, the study adds to the literature on in-depth crowd management in international research.

Supplementation efforts will have further implications. If contingency planning is used not only to respond to/recover from various special events but also to prepare for them, it can save a significant amount of time, especially in cases of emergencies ( IFRC, 2012: 5-12). Because contingency planning entails making multiple decisions beforehand regarding emergency resources, communication/coordination among stakeholders, and other technical responses, it will help to ensure the provision of emergency support during special events quickly enough. Hence, the amount of time spent on contingency planning is surely equal to the time saved in responding to disasters.

Stakeholders must fully utilize advanced technologies, including mobile technologies, Radio Frequency Identification (RFID), cloud computing, drone technology, smart cities, and others, to facilitate the supplementation process ( Yamin, 2019: 231-234). These can help in monitoring, managing, and controlling both special events and ordinary events. Further, officials’ slack attitude should also be improved.

Once contingency plans are developed for special events in local communities, those should be regularly tested and updated ( UN, 2008: 19-22). In general, local communities need to review developed contingency plans once a year. However, since the emergency environment has rapidly changed in the 21 ^st^ century regardless of national boundaries, stakeholders must gather together, evaluate contingency plans, and flexibly revise them as required. In addition, the updated contingency plans must be consistently and widely distributed to local residents via public communication channels. Further, the implementation of these plans must be kept very simple and realistic to avoid any delay in action ( Choularton, 2007: 25-30). To this end, the contents of contingency planning for special events should not be overly detailed but just appropriately detailed.

The level of public awareness of special events (or safety-first mindset) must be improved. The Itaewon tragedy happened despite KR having a history of 67 deaths in Busan in 1959, 31 deaths in Seoul in 1960, 12 deaths in Gwangju in 1965, and 11 deaths in Sangju in 2005 ( Lee, 2022a). Since the frequency of crowd crushes is much less than that of typhoons accompanied by floods or building fires, the Korean government reports or other research papers rarely include crowd crushes in the category of frequent disasters in KR. Hence, the level of disaster awareness on special events including crowd crushes is very limited.

Using the Itaewon crowd crush incident to carry out supplementation of plans will increase the situation awareness for crowd management ( Martella *et al.*, 2017: 377-378). The first responders did not know the danger of potential crowd crush in Itaewon, reflecting the poor level of situation awareness (i.e., too optimistic a scenario). Since the stakeholders lacked advanced disaster information, they could not make appropriate decisions regarding crowd crush management in time and thus failed to intervene appropriately. The level of situation awareness can be increased, in particular by utilizing human perception, as well as diverse scientific tools.

Supplementation does not mean that reliance on contingency planning fully guarantees the successful management of special events. Although sticking to contingency plans looks pretty effective for managing emergencies, the path to achieving them is still difficult. In a sense, even though contingency planning plays many roles in producing the extent of rigidity around special events (or allowing various participants to rigidly stick to the plan), it does not ensure the complete success of future emergency management, which includes various thorny issues.

Nonetheless, the supplementation principle must embody political rationality on a big scale ( Eriksson and McConnell, 2011: 95-97). In fact, politics around contingency planning and appropriate plans helps the elected officials and decision-makers to keep their reputation intact, delivers the image of controlled contingency for the public, and keeps the unhindered goal of national emergency management. Thus, by fully preparing for unexpected events, those high-ranking authorities may boost their political success in particular not only by managing special events but also by taking care of public fears of unforeseen catastrophes.

Emergency education, training, and exercise have to be fully employed to facilitate supplementation in KR. Many individuals and institutions fail to understand the exact differences between emergency planning for ordinary events and contingency planning for special events. Similarly, they are unfamiliar with the supplementation of the former with the latter. KR has to appropriately rely on these three ways for effective emergency management. While emergency education must provide generalized fundamentals for supplementation pursuit, emergency training and exercise can let participants practice detailed actions.

## Conclusion

This case study reviewed the Itaewon Halloween crowd crush in order to provide guidelines on how to prevent similar tragedies in KR or manage such a situation more efficiently to reduce human loss. The study identified previous studies, management challenges, appropriate alternatives, and implications. In particular, emergency planning for ordinary events and contingency planning for special events were compared and contrasted.

This study recommends that all stakeholders, including governments, businesses, voluntary organizations, and other local communities, must try to supplement emergency planning for ordinary events with contingency planning for special events in KR. Each stakeholder must work toward the establishment of Korean NRF, structural safety, expansion of voluntary activities, personal safety, and others sequentially. At the same time, all stakeholders must comply with coordination, time efficiency, regular updates, realistic approach, public awareness, political savvy, education and training, and others, to achieve full inclusion.

This paper is a pioneering study of Korean emergency management. However, a limitation of this research is that it is limited to KR. Since this study focuses on the Korean crowd crush only, the results of this paper might not have equal impacts on international researchers, despite the frequent reliance on principles of international emergency management.

Researchers are anticipated to further evaluate the Itaewon crowd crush in the near future. They may diversely interpret the national tragedy from the perspective of law, economy, psychology, computer science, road construction, and others. Besides, the impact of COVID-19 will continue to generate mass crowds and subsequent crowd crushes, irrespective of national borders. Therefore, international researchers can consider studying how to deal with the issue of potential crowd crush in their regions.

## Data Availability

DANS-EASY: Prisma Checklist, https://doi.org/10.17026/dans-zy4-8dzj ( Ha, 2023). This project contains the following extended data:
-
PRISMA_2020_checklist.pdf Data are available under the terms of the Creative Commons Zero “No rights reserved” data waiver (CC0 1.0 Public domain dedication).
