# Supplementary material for: Reviewing stakeholders during the Itaewon Halloween crowd crush, Korea 2022: Qualitative content analysis
Source: F1000Res. 2023 Nov 22;12:829. Originally published 2023 Jul 13. [Version 2] doi: 10.12688/f1000research.135265.2 (PMC10687381; doi:10.12688/f1000research.135265.2)
Supplement: Peer review history

# Reviewer report: Reviewer response for version 2

**DOI:** 10.5256/f1000research.159112.r224760

**Published:** 2023-12-11

**Authors:** Vineet Tiwari

**Affiliations:** 1 IIIT Allahabad, Allahabad, India

I have gone through the updated version and it seems OK to me and may be accepted. There are no comments further from my side.

Are the rationale for, and objectives of, the Systematic Review clearly stated?

Yes

Is the statistical analysis and its interpretation appropriate?

Not applicable

Are sufficient details of the methods and analysis provided to allow replication by others?

Partly

Are the conclusions drawn adequately supported by the results presented in the review?

Yes

Reviewer Expertise:

NA

I confirm that I have read this submission and believe that I have an appropriate level of expertise to confirm that it is of an acceptable scientific standard.

# Reviewer report: Reviewer response for version 2

**DOI:** 10.5256/f1000research.159112.r224759

**Published:** 2023-11-29

**Authors:** Corrado Rindone

**Affiliations:** 1 Universita degli Studi Mediterranea di Reggio Calabria, Reggio Calabria, Calabria, Italy

The new version improves the paper's quality.

Are the rationale for, and objectives of, the Systematic Review clearly stated?

Yes

Is the statistical analysis and its interpretation appropriate?

Yes

Are sufficient details of the methods and analysis provided to allow replication by others?

Partly

Are the conclusions drawn adequately supported by the results presented in the review?

No

Reviewer Expertise:

Transport planning. Evacuation planning

I confirm that I have read this submission and believe that I have an appropriate level of expertise to confirm that it is of an acceptable scientific standard.

# Reviewer report: Reviewer response for version 1

**DOI:** 10.5256/f1000research.148376.r209763

**Published:** 2023-11-08

**Authors:** Corrado Rindone

**Affiliations:** 1 Universita degli Studi Mediterranea di Reggio Calabria, Reggio Calabria, Calabria, Italy

The paper concerns the issue of crowd crushes. The specific event of  Itaewon Halloween crowd crush, in Korea is focused.

 The issue is very relevant and the paper is interesting.

 The current version presents some lacks in terms of literature review and methodology.

 In relation to the literature review, I suggest the author to consider the proposed emergency planning framework and in particular the preparedness with exercises.

 In this context, it is important to recall the evaluation methods for evacuation planning and the contribution of training action for risk reduction.

 As far as concerns the methodology, I suggest the authors to provide more details about analysis for aiding the potential reader to understand the proposed method.

 Finally, it is important to support the conclusions with some quantitative analysis and research's perspectives.

 Suggested references

 Russo et al (2014) Urban exposure: Training activities and risk reduction. WIT Transactions on Ecology and the Environment, 191, pp. 991-1001

Are the rationale for, and objectives of, the Systematic Review clearly stated?

Yes

Is the statistical analysis and its interpretation appropriate?

Yes

Are sufficient details of the methods and analysis provided to allow replication by others?

Partly

Are the conclusions drawn adequately supported by the results presented in the review?

No

Reviewer Expertise:

Transport planning. Evacuation planning

I confirm that I have read this submission and believe that I have an appropriate level of expertise to confirm that it is of an acceptable scientific standard, however I have significant reservations, as outlined above.

## References

[1] : Urban exposure: Training activities and risk reduction. *WIT Transactions on Ecology and the Environment* .2014;191:991-1001

# Response

**Published:** 2023-11-19

**Authors:** Kyoo-Man Ha

**Corrado Rindone**

 Universita degli Studi Mediterranea di Reggio Calabria, Reggio Calabria, Calabria, Italy

 The paper concerns the issue of crowd crushes. The specific event of  Itaewon Halloween crowd crush, in Korea is focused.

 The issue is very relevant and the paper is interesting.

 Response: Thank you very much for your general comments.

 The current version presents some lacks in terms of literature review and methodology.

 Response: O.K. This was duly noted.

 In relation to the literature review, I suggest the author to consider the proposed emergency

 planning framework and in particular the preparedness with exercises.

 Response: I added a paragraph to the section of Literature review by referring to (Russo and Rindone, 2014).

 In this context, it is important to recall the evaluation methods for evacuation planning and the

 contribution of training action for risk reduction.

 Response: As a similar token, I addred related contents to above paragraph .

 As far as concerns the methodology, I suggest the authors to provide more details about analysis for aiding the potential reader to understand the proposed method.

 Response: Despite the lack of your comments, I made all efforts to provide more detaila on the methodology.

 Finally, it is important to support the conclusions with some quantitative analysis and research's

 perspectives.

 Response: I added related contents as a strength of this article to the section of Conclusion.

 Suggested references

 Russo et al (2014) Urban exposure: Training activities and risk reduction. WIT Transactions on Ecology and the Environment, 191, pp. 991-1001

 Response: As mentioned earlier, I surely referred to this article in the revised manuscript.

 References

 1. Russo F, Rindone C: Urban exposure: Training activities and risk reduction. WIT Transactions on Ecology and the Environment. 2014; 191: 991-1001

 Are the rationale for, and objectives of, the Systematic Review clearly stated?

 Yes

 Are sufficient details of the methods and analysis provided to allow replication by others?

 Partly

 Is the statistical analysis and its interpretation appropriate?

 Yes

 Are the conclusions drawn adequately supported by the results presented in the review?

 No

*Competing Interests*: No competing interests were disclosed.

*Reviewer Expertise:* Transport planning. Evacuation planning

# Reviewer report: Reviewer response for version 1

**DOI:** 10.5256/f1000research.148376.r214388

**Published:** 2023-11-06

**Authors:** David McEntire

**Affiliations:** 1 Utah Valley University, Orem, Utah, USA

This is an important study on a topic that has not received ample attention from scholars and practitioners. In this sense, the paper is timely and relevant.

 For the most part, I like the article and believe it makes important contributions to the literature. Methods are adequate.

 There are a few challenges:

1.  More information can be given about the crowd crush in the introduction and in order to set the context of the paper.
2. The literature review needs some work. Some concepts are mentioned but they are not explained: e.g., interruption, flight, craze, open area, hidden area, blind area, and unknown area.
3. The literature review section could be reorganized to improve flow and ease of reading. For instance, the desire to meet in person could be put earlier in the paper or at the end of the literature review. The paragraph regarding Almutairi's work go go earlier, and then all of the findings could be mentioned about crowd crushes. Then, the discussion about how to deal with them can occur.
4. Finally, the title might be misleading. The paper is not necessarily about the crowd crush per se, but about what governments, businesses, voluntary organizations, and other community groups did before, during and after.  The title could reflect this priority.

Overall, I like what I read. Just a few suggestions to make it better.

Are the rationale for, and objectives of, the Systematic Review clearly stated?

Partly

Is the statistical analysis and its interpretation appropriate?

Not applicable

Are sufficient details of the methods and analysis provided to allow replication by others?

Yes

Are the conclusions drawn adequately supported by the results presented in the review?

Yes

Reviewer Expertise:

Emergency management, homeland security.

I confirm that I have read this submission and believe that I have an appropriate level of expertise to confirm that it is of an acceptable scientific standard, however I have significant reservations, as outlined above.

# Response

**Published:** 2023-11-19

**Authors:** Kyoo-Man Ha

**David McEntire**

 Utah Valley University, Orem, Utah, USA

 This is an important study on a topic that has not received ample attention from scholars and

 practitioners. In this sense, the paper is timely and relevant.

 For the most part, I like the article and believe it makes important contributions to the literature.

 Methods are adequate.

 Response: Thank you very much for giving me another opportunity.

 There are a few challenges:

 1. More information can be given about the crowd crush in the introduction and in order to

 set the context of the paper.

 Response: I added a paragraph on the crowd crush to the beginning of Introduction.

 2. The literature review needs some work. Some concepts are mentioned but they are not

 explained: e.g., interruption, flight, craze, open area, hidden area, blind area, and unknown

 area.

 Response: I explained them all.

 3. The literature review section could be reorganized to improve flow and ease of reading. For instance, the desire to meet in person could be put earlier in the paper or at the end of the

 literature review. The paragraph regarding Almutairi's work go go earlier, and then all of

 the findings could be mentioned about crowd crushes. Then, the discussion about how to

 deal with them can occur.

 Response: I tried to reflect your comments to the section of Literature review, despite the lack of your explanatiion. Such as, I moved “the desire to meet..” to the section of Introduction. After that, I re-arranged “Almutairi’s work..,” “all of the findings,” and “discussion about how to deal with..” sequentially.    

 4. Finally, the title might be misleading. The paper is not necessarily about the crowd crush per se, but about what governments, businesses, voluntary organizations, and other

 community groups did before, during and after. The title could reflect this priority.

 Response: I revised the title of paper.

 Overall, I like what I read. Just a few suggestions to make it better.

 Response: Once again, thank you very much for yoru comments.

 Are the rationale for, and objectives of, the Systematic Review clearly stated?

 Partly

 Are sufficient details of the methods and analysis provided to allow replication by others?

 Yes

 Is the statistical analysis and its interpretation appropriate?

 Not applicable

 Are the conclusions drawn adequately supported by the results presented in the review?

 Yes

*Competing Interests: *No competing interests were disclosed.

*Reviewer Expertise: *Emergency management, homeland security.

# Reviewer report: Reviewer response for version 1

**DOI:** 10.5256/f1000research.148376.r188677

**Published:** 2023-08-03

**Authors:** Vineet Tiwari

**Affiliations:** 1 IIIT Allahabad, Allahabad, India

The author has done a good case study regarding Itaewon Halloween crowd crush but some points needs to be included in this study.

1. The author focused on the role of four stakeholders but the importance of other factors cannot be ignored like Capacity assessment of organizing agency **, **Crowd behaviour and psychology, Role of Information and Communication Technology (ICT) etc.
2. Separate Traffic management and comprehensive mobility plan for Emergency planning and contingency planning.
3. On which criteria Emergency planning and contingency planning should be differentiated?
4. What other countries responded in similar cases should be illustrated in the paper to make a comparative study.
5. The actual cause of Itaewon Halloween crowd crush is not described in this study and what was the Government's planning to organize such a big events.
6. The history of Itaewon Halloween program should be given with emphasis on earlier accidents if any?
7. What would be the coordination mechanism for all four stakeholders?
8. Results section is not very clear.

Suggestions regarding recent research:

1. https://doi.org/10.1111/bjso.12666
2. https://doi.org/10.1016/j.physa.2023.129002
3. https://doi.org/10.1017/S1049023X2300225X
4. https://doi.org/10.1007/s40009-022-01114-w

Are the rationale for, and objectives of, the Systematic Review clearly stated?

Yes

Is the statistical analysis and its interpretation appropriate?

Not applicable

Are sufficient details of the methods and analysis provided to allow replication by others?

Partly

Are the conclusions drawn adequately supported by the results presented in the review?

Yes

Reviewer Expertise:

Crowd management events like largest mass gathering event in India: Kumbh Mela

I confirm that I have read this submission and believe that I have an appropriate level of expertise to confirm that it is of an acceptable scientific standard, however I have significant reservations, as outlined above.

## References

[1] : Beyond 'stampedes': Towards a new psychology of crowd crush disasters. *Br J Soc Psychol* .2023; 10.1111/bjso.12666 10.1111/bjso.12666 37387244

[2] : An extended model for crowd evacuation considering crowding and stampede effects under the internal crushing. *Physica A: Statistical Mechanics and its Applications* .2023;625: 10.1016/j.physa.2023.129002 10.1016/j.physa.2023.129002

[3] : Survey of Crowd Crush Disasters and Countermeasures. *Prehospital and Disaster Medicine* .2023;38(S1) : 10.1017/S1049023X2300225X 10.1017/S1049023X2300225X

[4] : Crowd Management and Strategies for Security and Surveillance During the Large Mass Gathering Events: The Prayagraj Kumbh Mela 2019 Experience. *National Academy Science Letters* .2022;45(3) : 10.1007/s40009-022-01114-w 263-273 10.1007/s40009-022-01114-w PMC898910435431383

# Response

**Published:** 2023-11-19

**Authors:** Kyoo-Man Ha

**Vineet Tiwari**

 IIIT Allahabad, Allahabad, India

 The author has done a good case study regarding Itaewon Halloween crowd crush but some

 points needs to be included in this study.

 Response: Thank you very much for your comments, first of all.

 1. The author focused on the role of four stakeholders but the importance of other factors

 cannot be ignored like Capacity assessment of organizing agency **, **Crowd behaviour and

 psychology, Role of Information and Communication Technology (ICT) etc.

 Response: About capacity assessment of organizing agency, I already mentioned that there was no official organizing agency around the Itaewon crowd. Nonetheless, I added the importance of assessing organizing agency’s capacity to the section of Implicaitons. On crowd behavior and psychology, I further explained the topic (Barr et al., 2023) in the section of Literature review. Regarding ICT, I further expanded its importance (Wang, 2023) in the section of Implications.

 2. Separate Traffic management and comprehensive mobility plan for Emergency planning

 and contingency planning.

 Response: I added appropriate contents on related differences (Kanaujiya and Tiwari, 2022) to the section Literature review.  

 3. On which criteria Emergency planning and contingency planning should be differentiated?

 Response: I further explained related criteria in the section of Literature review.

 4. What other countries responded in similar cases should be illustrated in the paper to make

 a comparative study.

 Response: I further explained similar cases in other countries in the section of Literature review.

 5. The actual cause of Itaewon Halloween crowd crush is not described in this study and what

 was the Government's planning to organize such a big events.

 Response: I am sorry, but I already provided related answers. The actual cause was the lack of contingency planning in Korea. Similarly, Korean government did not do anything with planning to organize such a big event.

 6. The history of Itaewon Halloween program should be given with emphasis on earlier

 accidents if any?

 Response: No, it did not have any earlier accident in terms of history.

 7. What would be the coordination mechanism for all four stakeholders?

 Response: I added a paragraph on coordination mechanism to the section of Implications.

 8. Results section is not very clear.

 Resposne: Despite the lack of your comments, I did my best to read and then improve related contexts.

 Suggestions regarding recent research:

 Response: I referred to all four articles in my revised manuscript.

 1. https://doi.org/10.1111/bjso.12666

 2. https://doi.org/10.1016/j.physa.2023.129002

 3. https://doi.org/10.1017/S1049023X2300225X

 4. https://doi.org/10.1007/s40009-022-01114-w

 References

 1. Barr D, Drury J, Butler T, Choudhury S, et al.: Beyond 'stampedes': Towards a new psychology of crowd crush disasters. *Br J Soc Psychol*. 2023. PubMed Abstract | Publisher Full Text

 2. Chen C, Lu T, Jiao W, Shi C: An extended model for crowd evacuation considering crowding and stampede effects under the internal crushing. *Physica A: Statistical Mechanics and its Applications*.

 2023; **625**. Publisher Full Text

 3. Wang S: Survey of Crowd Crush Disasters and Countermeasures. *Prehospital and Disaster*

*Medicine*. 2023; **38 **(S1). Publisher Full Text

 4. Kanaujiya A, Tiwari V: Crowd Management and Strategies for Security and Surveillance During the Large Mass Gathering Events: The Prayagraj Kumbh Mela 2019 Experience. *National Academy* *Science Letters*. 2022; **45 **(3): 263-273 Publisher Full Text

 Are the rationale for, and objectives of, the Systematic Review clearly stated?

 Yes

 Are sufficient details of the methods and analysis provided to allow replication by others?

 Partly

 Is the statistical analysis and its interpretation appropriate?

 Not applicable

 Are the conclusions drawn adequately supported by the results presented in the review?

 Yes

*Competing Interests: *No competing interests were disclosed.

*Reviewer Expertise: *Crowd management events like largest mass gathering event in India: Kumbh Mela
